# Carrier-free supramolecular nanoassemblies of pure LSD1 inhibitor for effective anti-tumor therapy

**DOI:** 10.3389/fchem.2022.1012882

**Published:** 2022-09-30

**Authors:** Boao Li, Xiangyu Zhang, Jibin Li

**Affiliations:** ^1^ Department of Colorectal Surgery, Liaoning Cancer Hospital, Shenyang, China; ^2^ State Key Laboratory of Natural and Biomimetic Drugs, School of Pharmaceutical Sciences, Peking University, Beijing, China

**Keywords:** drug delivery, supramolecular nanoassemblies, therapeutic efficiency, systemic toxicity, drug-like compound

## Abstract

The LSD1 protein is an oxidase that regulates protein methylation, which regulates gene expression and triggers tumors. Previously, inhibiting LSD1 has been found to be an effective treatment strategy for opposing tumors caused by overexpression of LSD1. Our recent study found that compound **17i** was a suitable LSD1 inhibitor with potential anti-tumor activity. However, its extremely insoluble nature limits further validation of its anti-tumor activity at the clinical level. In this study, a unique carrier-free supramolecular nanoassemblies of pure compound **17i** is expected to enhance therapeutic efficacy. Aqueous-insoluble compound **17i** was mixed with a small quantity of DSPE-PEG_2000_ into an organic solvent and was prepared as nanoassemblies in water via the one-step nanoprecipitation method. The **17i** nanoassemblies have a similar effect on its cytotoxicity when compared with **17i** solution *in vitro*. Importantly, the PEGylated **17i** nanoassemblies exhibit significant superiorities over **17i** solutions in therapeutic efficiency, anti-tumor immune response and systemic toxicity in BALB/c mice bearing CT-26 colorectal tumors. We envision that the fabrication of pure drug nanoassemblies offers an efficient platform for reforming the undesirable characteristics of drug-like compounds to potentiate the anti-tumor therapeutic effect.

## Introduction

**FIGURE 1:**
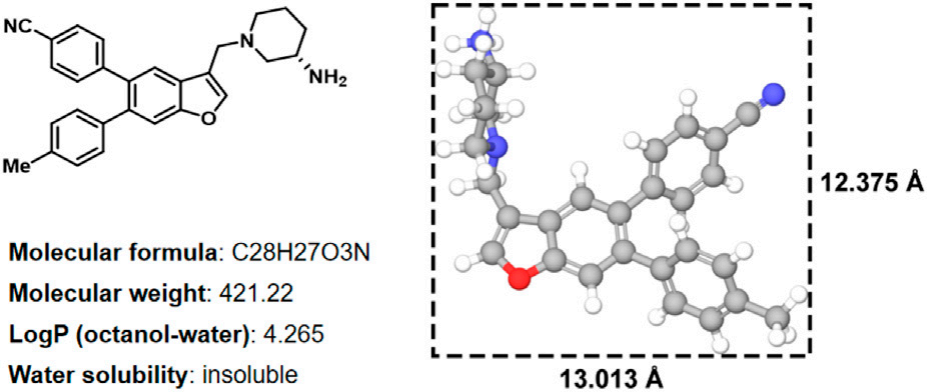
Two-dimensional chemical structure, physicochemical properties, three-dimensional structures and sizes of compound **17i** (Å, angstrom).

In histone lysine specific demethylase 1 (LSD1), the methyl groups are removed from the lysine residues (H3K4 and H3K9) by an oxidative enzyme (Shi et al., 2004; Suzuki and Miyata, 2011; Schmitt et al., 2013; Sorna et al., 2013; Tortorici et al., 2013; Zheng et al., 2013). As an epigenetic regulator, gene expression and cancer initiation are influenced by LSD1 (Ma et al., 2015; Zheng et al., 2015; Vianello et al., 2016). Therefore, inhibiting LSD1 is an effective strategy for anti-tumor treatment (Wu et al., 2016; Mould et al., 2017). In our previous study, various LSD1 inhibitors were reported (Wang X. et al., 2020; Zhang et al., 2021), in which the compound **17i** (Figure 1) (IC_50_ = 0.065 *μ*M) exhibited a significant effect on target LSD1. The solubility of compound **17i** in organic solvents was good. However, it has been tested that **17i** is almost insoluble in water. This impeded further validation of its anti-tumor activity at the clinical level.

The wide application of nanotechnology in the medical field has significantly enriched the delivery strategies of anti-tumor drugs (Sun M. et al., 2019; Sun et al., 2022). Especially, the construction of a novel nanodrug delivery system can significantly improve the druggability of chemotherapeutics, prolong the systemic circulation time and enhance tumor-specific accumulation by increasing permeability and retention (EPR) effect, thus augmenting the anti-tumor effect and reduce the side effects (Sun B. et al., 2019; Wang Q. et al., 2020; Yang et al., 2020; Zhang et al., 2020; Zhao et al., 2021). Herein, to solve the dilemma of clinical transformation of compound **17i**, we put forward a fascinating nanoassembly based on compound **17i** for effective anti-tumor therapy. Firstly, we confirm tight interactions between LSD1 and compound **17i** through molecular docking and molecular dynamic simulation, elaborating that compound **17i** had effective inhibitory activity for LSD1. The supramolecular nanoassemblies of **17i** were fabricated by a one-step nanoprecipitation approach. A small amount of 1,2-distearoyl-sn-glycero-3-phosphoethanolamine-N-[methoxy (polyethyleneglycol)-2000] (DSPE-PEG_2000_) (Supplementary Figure S3) was attached on the surface to improve surface hydrophilicity of nanoassemblies and extend the period of blood circulation. The PEGylated **17i** nanoassemblies exhibited comparable cytotoxicity when compared with **17i** solution *in vitro*, but showed particular superiorities in terms of efficient anti-tumor therapy and anti-tumor immune response and less side effect in BALB/c mice bearing CT-26 tumors. As far as we are aware, this is the first time pure LSD1 inhibitor has been fabricated into nanoassemblies without the addition of carrier excipients. Such a potent nanoplatform holds promising clinical application prospects for drug-like compounds.

## Materials and methods

### Materials

Compound **17i** was self-prepare. 2-(4-Amidinophenyl)-6-indolecarbamidine dihydrochloride (DAPI) and 3-(4,5-Dimthyl-2-thiazolyl)-2,5-dipphenyl-2H-terazolium bromide (MTT) were obtained from Dalian Meilun Biotech Co., Ltd. (Dalian, China). Cell culture media RPMI 1640, penicilline-streptomycin, and fetal bovine serum were available from GIBCO, (Carlsbad, United States). 1,2-distearoyl-sn-glycero-3-phosphoethanolamine-N-[methoxy (polyethyleneglycol)-2000] (DSPE-PEG_2000_) was purchased from Shanghai Advanced Vehicle Technology Co., Ltd. All other chemical components and solvents applied in this study are of analytical grade.

### Molecular docking

To predict the binding mode of the target molecule with the binding site, we performed molecular docking using Glide 9.7 module (Friesner et al., 2004; Friesner et al., 2006), which the protein structure PDB 5YJB (residues 172–833) used for docking. The inhibitor **17i** was docked into the binding pocket of LSD1 using the standard precision module to get initial binding predictions and docking sores.

### MD simulation

The 100 ns MD simulations were carried out of LSD1-**17i** complex by using Desmond v3.8 (Wang et al., 2019). This system is dissolved in a cubic box (8 Å ✕30 Å ✕8 Å) with a simple point charge (SPC) water, adding an appropriate amount of Na^+^ counter ions to achieve neutralization. Based on the OPLS-2005 force field, the energy of the system was minimized. Finally, the 100 ns MD simulations were performed in NPT ensemble. Other parameters are default.

### Preparation of supramolecular 17i nanoassemblies

Nanoassemblies based on pure **17i** compound were prepared by the one-step nanoprecipitation method. 8 mg **17i** was dissolved in 1 ml methanol to acquire **17i** methanol solution. Then, 500 μL mixtures were dropped slowly into the 2 ml aqueous solution under the stirring for 5 min. Following that, methanol was removed from the colloidal solution in a vacuum at 37°C. The PEGylated **17i** nanoassemblies were prepared in above protocol using a mixed methanol solution of **17i** and DSPE-PEG_2000_ (15%, w/w). The prepared nanoassemblies would be stored at 4°C. In addtion, the **17i** reagents were dissolved in 0.5 ml acetonitrile. Then, the mother liquor was diluted with PBS (1:9), prepared as the **17i** solution.

### Characterization of supramolecular 17i nanoassemblies

The dynamic light scattering particle size of **17i** nanoassemblies was measured through a Zetasizer (Malvern Co., UK). The prepared nanoassemblies were diluted with phosphate buffer solution (PBS) and the particle size was measured three times. The **17i** nanoassemblies were diluted 1/20 with deionized water and dropped onto a copper mesh (300 mesh) covered by carbon film. After natural drying, negative staining was performed with 2% phosphotungstate acid. Transmission electron microscopy (TEM) (Hitachi, HT7700, Japan) was used to observe the appearance and morphology of **17i** nanoassemblies.

Binding conformation and binding energy of two molecules of **17i** together was calculated with molecular docking. Docking was performed using Glide module in Schrödinger. One molecule of **17i** was selected as the receptor and receptor grid was set to cover the whole receptor molecule, and then another molecule of **17i** was docked onto **17i** receptor, and binding energy were calculated.

Molecular dynamic (MD) simulation was performed with Materials Studio Program (Accelrys Inc.). First, amorphous cell module was used to construct molecular aggregation models in which 16 **17i** molecules and 11170 water molecules were put into cubic box with side length equals 7ns. Then 50000 steps energy minimization were performed followed with 50 ns molecular dynamic with compass force field in temperature of 298K, pressure of 1.01325 bar and NPT ensemble. Root mean square derivation (RMSD) value were calculated using forcite module.

### Simulation study of assembly

Computational simulations of intermolecular interactions between **17i** molecules were performed. The two-dimensional (2D) structure of **17i** was established by Marvin sketch software, and the three-dimensional (3D) structure of **17i** was optimized by Sybyl 6.9.1 software package. In the previous work, we have introduced the runtime simulation environment and other method parameters in detail.

### Cell viability

CT-26 cells were cultured with RPMI-1640 cells containing 10% FBS and 100 μg/ml 3 × 10^3^ cells were cultured in 96-well plates for 12 h to evaluate the cellular viability of **17i** nanoparts. Next, the medium was replaced with different concentrations of **17i** solutions and **17i** nanocomposites containing 10% alcohol.

24 or 48 h later, MTT solution (1 mg/ml) was placed in an incubator for further incubation for about 4 h. Drained from supernatant, DMSO was then added to each well, and the purple crystals were fully dissolved by slowly shaking for 5 min. The absorbance value of each well was measured at 492 nm by the multifunctional microplate analyzer.

### Animal studies

All animal protocols were evaluated and approved by the Animal Laboratory Ethics Committee of the Liaoning Cancer Hospital. The BALB/c mice bearing CT-26 tumors were established. PBS, **17i** solution and PEGylated **17i** nanoassemblies (20 mg/kg **17i**) were intraperitoneal-injected into the mice separately. The drug was administered every other day for five times, and the tumor volume was measured and weighed daily. On the last day of the efficacy trial, animals were killed, and tumor tissues were isolated, weighed, and photographed to compare the effects of different preparations on tumor growth.

### Flow cytometry analysis

Tumor tissues after different treatments were extracted and collected from CT-26-bearing BALB/c mice. Tumor tissue were conducted to prepare a single-cell suspension. Then, the cells were stained with fluorescence-labeled antibodies CD3, CD4 and CD8 in compliance with the instructions of manufacturers. The proportion of stained cells from tumor tissues were measured using flow cytometry and data were analyzed using FlowJo software.

### Statistical analysis

Statistical analysis was carried out using Graphpad Prism software. All data results were showed as mean ± standard deviation (SD). Student’s T-test was used to analyze differences between groups. The *p* < 0.05 was deemed statistically significant (Sun B. et al., 2019; Sun M. et al., 2019; Wang Q. et al., 2020; Sun et al., 2022).

## Results

### Computational simulation studies

**FIGURE 2:**
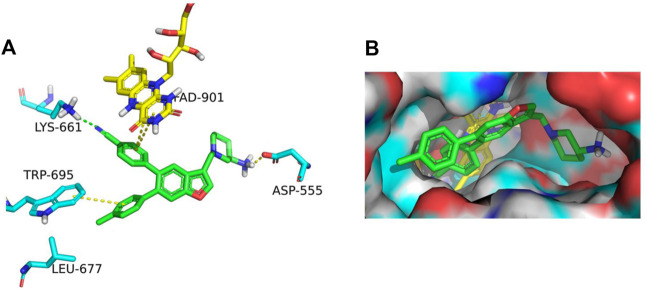
**(A)** In LSD1, **17i** (green) binds to its active site. **(B) 17i** represents the binding surface of the LSD1 pocket (PDB: 5YJB).

The binding affinity of inhibitor **17i** in LSD1 (PDB code 5YJB) was first evaluated using a combined docking and molecular dynamics protocol. Figure 2 illustrates the possible binding schema between **17i** and LSD1 was predicted by glide 9.7 software with a high binding affinity (SP score = -9.109 kcal/mol), consisting of its bioactivity (IC_50_ = 65 nM).

**FIGURE 3:**
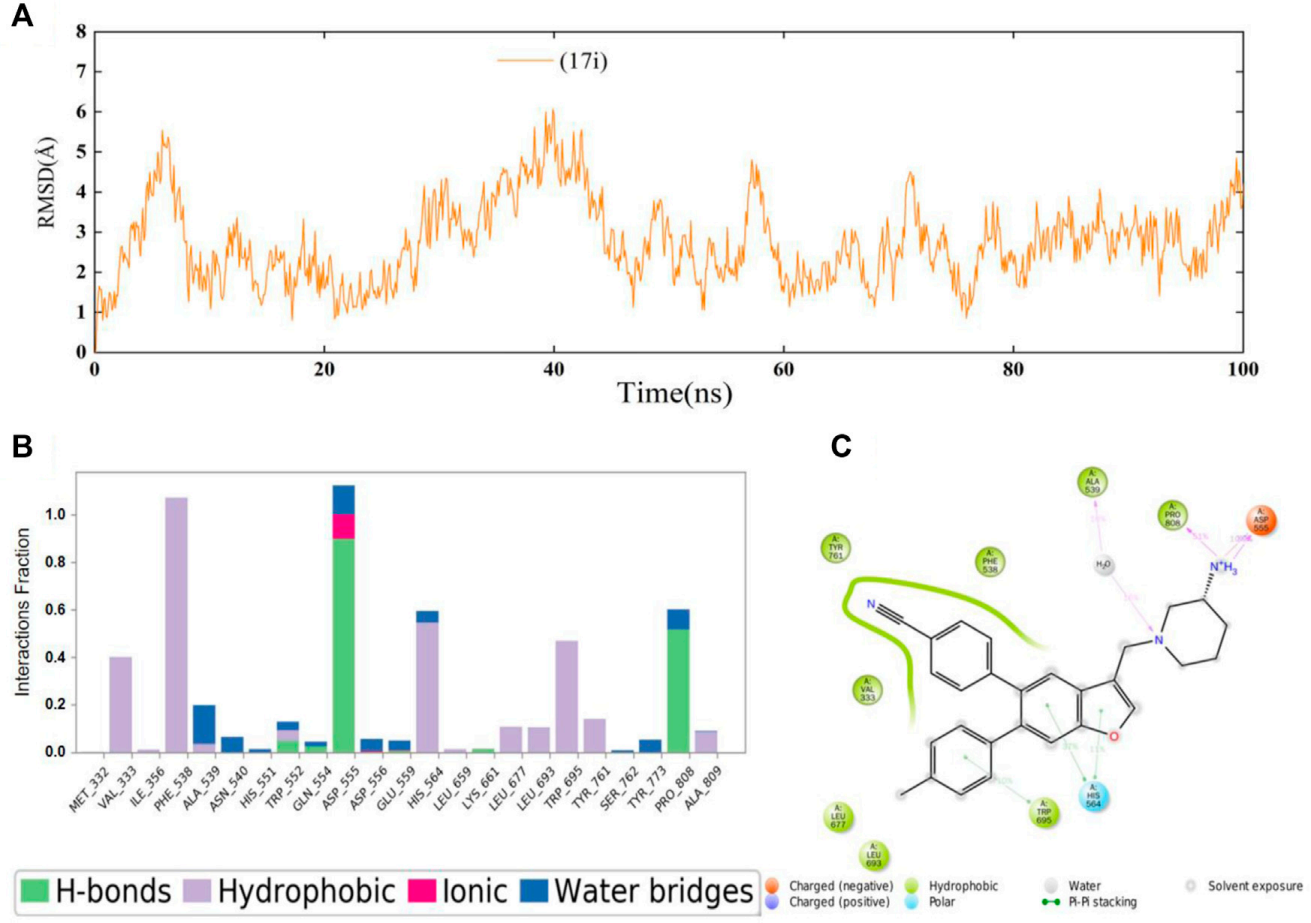
**(A)** Time evolution of the RMSD value of compound **17i** analyzed by 100 ns molecular dynamic (MD) simulations. **(B)** Statistical protein-ligand contacts of compound **17i** and **(C)** Two-dimensional interactions of compound **17i** during the whole MD simulations.

Next, 100 ns MD simulations of the protein-ligand complex (LSD1 protein and compound **17i**) were performed to predefined binding modes using Desmond v3.8. As depicted in Figure 3A, the LSD1-**17i** complex reached equilibrium at about 80 ns and the fluctuation of RMSD values (Å) was found to be 2–4 Å, indicating that compound **17i** was stabilized favorably with the active site during the binding process. Meanwhile, the contributions of amino acid interactions were also analyzed during the MD simulation in Figures 3B,C. Hydrogen bonds were formed between the piperidine-3-amine moiety of **17i** and Asp555 and Pro808, accounting for 100 and 51% respectively. The nitrogen atom on the piperidine ring also interacted with Ala539 through water molecules to form the hydrogen bond, counting for 16%. Surprisingly, the Lys661-mediated hydrogen bond interaction was weak (less than 10%). There was little that Lys661 contributed to the activity of the compound **17i** during the binding process. In addition, some new residues not determined by molecular docking were observed, such as His564. It was positioned in proximity of compound **17i** and participated in π-π stacking interactions with the ring of benzofuran ring.

### Preparation and characterization of 17i nanoassemblies

For the fabrication of **17i** nanoassemblies, one-step nanoprecipitation was used. The hydrophobic **17i** molecules spontaneously assembled into uniform **17i** nanoassemblies formed without the aid of any carrier excipients. We constructed PEGylated **17i** nanoassemblies with a small quantity DSPE-PEG_2000_ (15 wt%). Obviously, nanoassemblies composed of **17i** molecules were obviously the main components, and PEGylated **17i** nanoassemblies loaded drugs more than 80 wt% efficiently.

**FIGURE 4:**
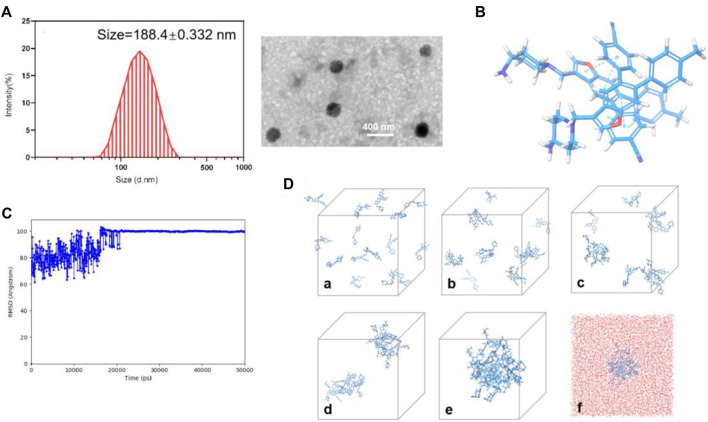
**(A)** Particle size distribution and TEM images of PEGylated **17i** nanoassemblies. **(B)** Docking diagram of the assembly mechanism of compound **17i**. **(C)** Root Mean Square Deviation (RMSD) values of the nanoassembly system. **(D)** The aggregation process of **17i** in water at **(a)** 0 ns, **(b)** 2.5 ns, **(c)** 6.1 ns, **(d)** 14 ns, **(e)** 50 ns and **(f)** 50 ns contained water beads (red beads).

**17i** molecules themselves were the main component of nanoassemblies, and the drug loading efficiency of the PEGylated **17i** nanoassemblies was more than 80 wt%. The hydrated particle size of the nanoassemblies by dynamic light scattering (DLS) was 188.4 ± 0.332 nm. The image showed the irregular spheres and a particle size of <200 nm in dehydrated diameter, as determined by transmission electron microscopy (TEM) (Figures 4A,B). The critical aggregation concentration (CAC) of **17i**-based nanoassemblies was equal to 1 μg/ml compound **17i**. Subsequently, the assembly mechanisms of pure compound **17i** were then evaluated using molecular docking. Multiple intermolecular interactions led to the formation of hydrogen bonds, hydrophobic interactions, and stacking interactions in this nanosystem (Figure 4C). It was assumed that the hydrophobic interactions among compound **17i** molecules could drive to gather, and the hydrophilic amino groups in **17i** molecules were exposed and coexisted stably with water by hydrogen bonds.

**FIGURE 5:**
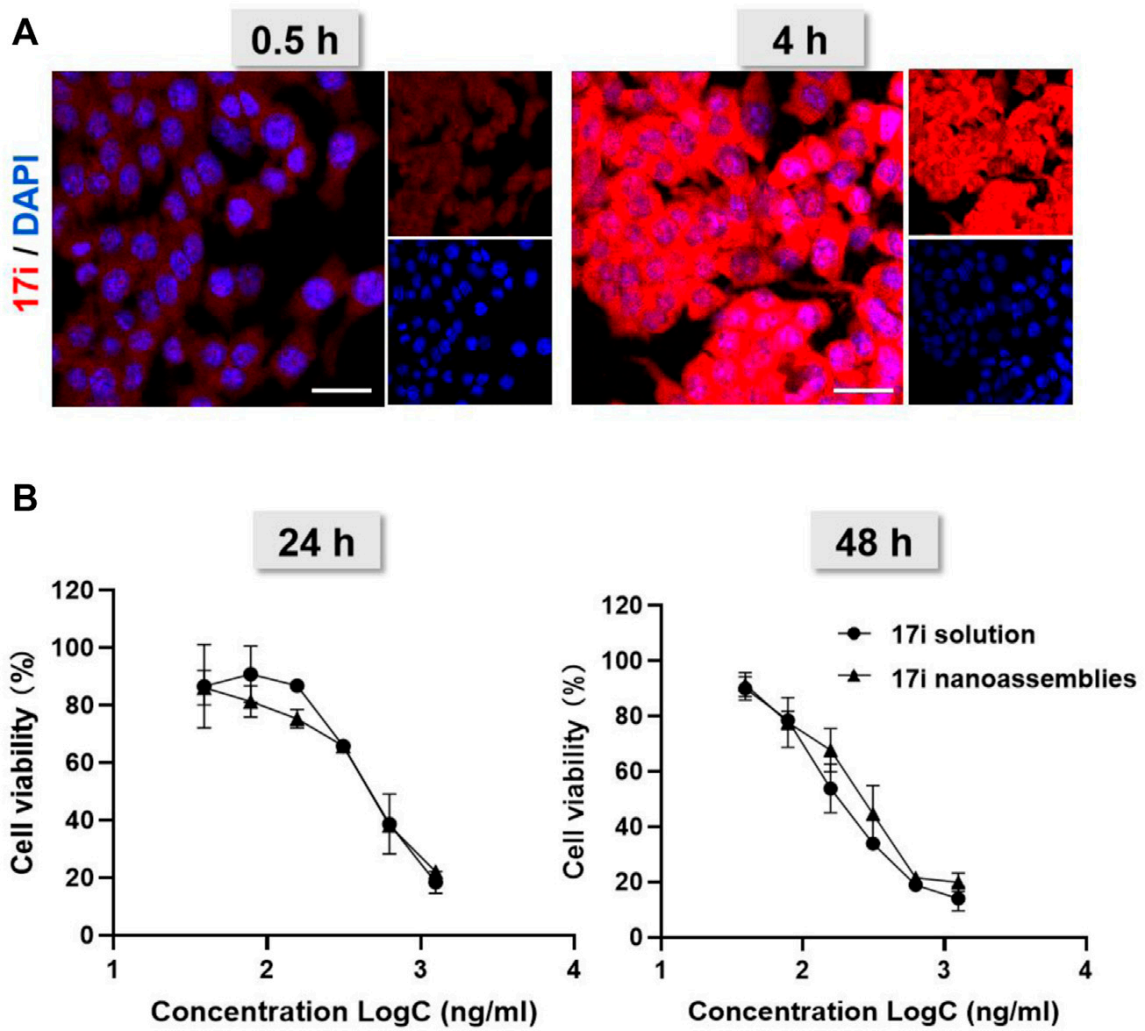
**(A)** Cellular uptake: confocal laser scanning microscopy (CLSM) images of CT-26 cells after being treated for 0.5 and 4 h with **17i** nanoassemblies. Scale bar = 20 μm. **(B)** Viability of CT-26 cells after treated with various concentrations of **17i** solution and **17i** nanoassemblies for 24 and 48 h, respectively (n = 3).

Next, we investigated the cellular uptake of PEGylated **17i** nanoassemblies by measuring the amount of **17i** after incubation with CT-26 murine colorectal tumor cells. Figure 5A shows that, with the passage of time, **17i** nanoassemblies treated with PEGylated nanoparticles produced stronger red intracellular fluorescence. In addition, a MTT assay was conducted to determine the cytotoxicity of **17i** solution and PEGylated **17i** nanoassemblies *in vitro* (Figure 5B). For CT-26 cells, the cytotoxicity of PEGylated **17i** nanoassemblies was comparable to that of **17i** compound solutions, demonstrating that **17i** nanoassemblies had a negligible effect on its *in vitro* cytotoxicity. The wound healing assay and apoptosis induction was also used to investigate the anti-cancer activity *in vitro* (Supplementary Figure S4). Similar to results of the cytotoxicity experiment, the anti-cancer ability of **17i** nanoassemblies make it a potent candidate for further *in vivo* evaluation.

### *In vivo* antitumor studies

**FIGURE 6:**
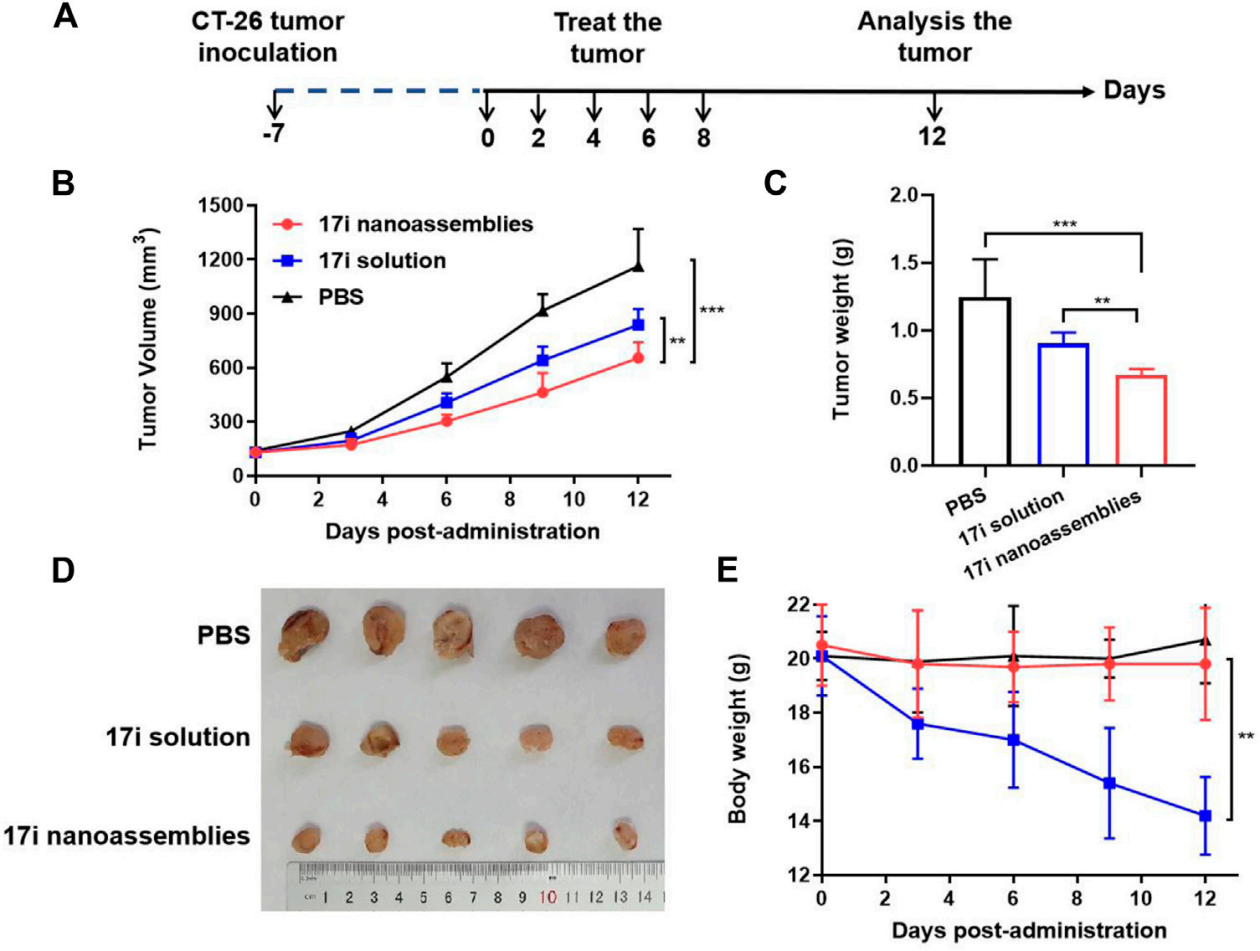
*In vivo* anti-tumor efficacy of **17i** solution and PEGylated **17i** nanoassemblies against CT-26 tumors. **(A)** Therapeutic protocol on mouse CT26 subcutaneous tumor xenograft. **(B)** Tumor volume growth curve after different treatments. **(C)** Tumor weight **(G)** of mice after 12 days in different treatments (n = 5). **(D)** After repeated administration, excised tumors of different groups are shown. **(E)** Changes in body weight of mice during different treatments (n = 5).

**FIGURE 7:**
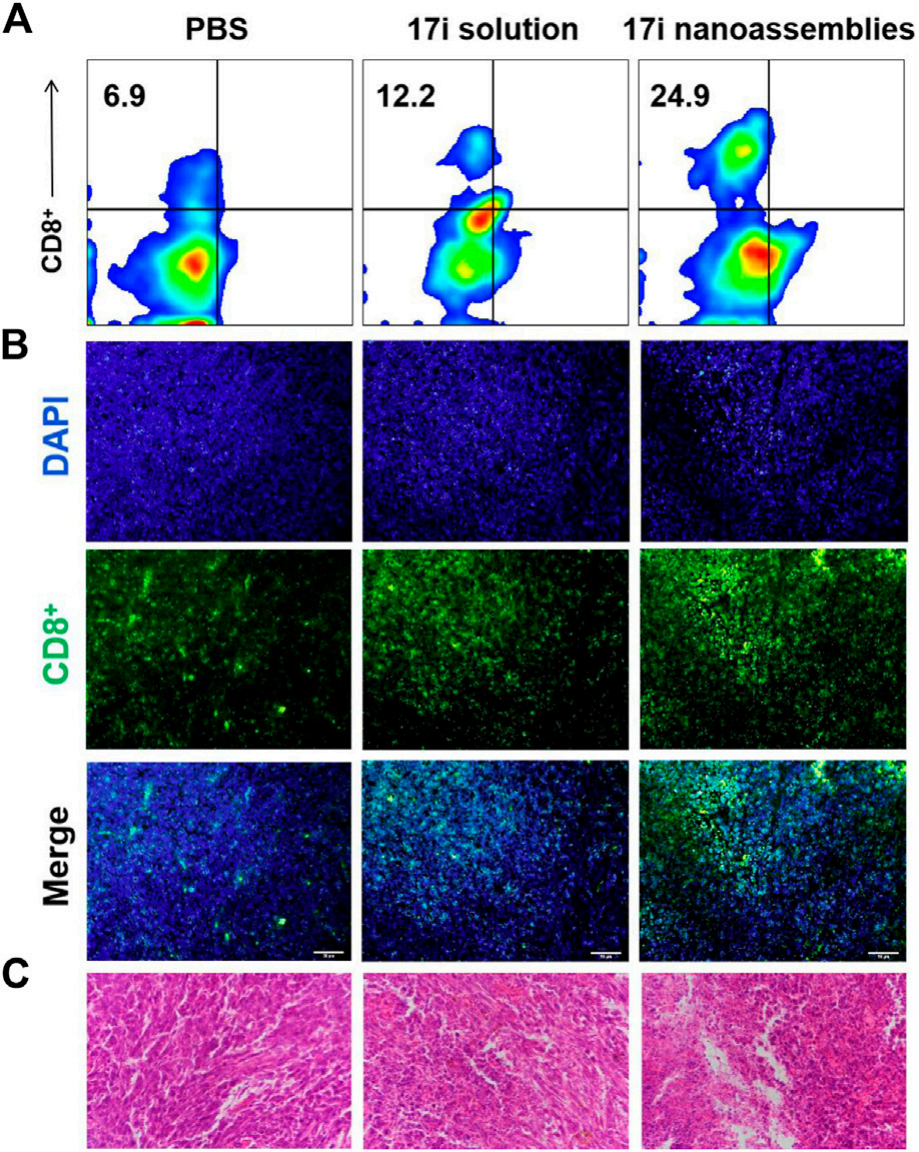
**(A)** Flow cytometry, **(B)** immunofluorescence and **(C)** H&E stained images of tumor slices. The nuclei were stained with DAPI (blue) and CD8 antibody (green). Scale bar = 50 μm.

This part evaluated the anti-tumor activity of CT-26 *in vivo*. PBS, **17i** solution and PEGylated **17i** nanoassemblies (20 mg kg^−1^ of **17i**) were treated by intraperitoneal administration for a total of five times. As shown in Figure 6, the PBS group could not inhibit the rapid growth of the tumor. In contrast, both **17i** solution and PEGylated **17i** nanoassemblies had an anti-tumor effect, and the tumor growth rate was significantly slowed down. The PEGylated **17i** nanoassemblies had a much stronger anti-tumor effect than the **17i** solution. Like the intraperitoneal administrated model, the anti-tumor activity of **17i** nanoassemblies in the intravenous administrated model was better than the other groups (Supplementary Figure S5). In addition, as illustrated in Figure 7, In immunofluorescence staining and flow cytometry, C57 mice bearing CT-26 were significantly infiltrated by CD8^+^ T cells in tumor regions after receiving PEGylated **17i** nanoassemblies. It is speculated that the appropriate particle size of PEGylated **17i** nanoassemblies was more conducive to drugs enrichment in the tumor site *via* the EPR effect. An enhanced anti-tumor efficacy was demonstrated by the presence of enriched **17i** at tumor sites, resulting in an increased number of CD8^+^ T cells infiltrating the tumor site. The PEGylated nanoassemblies showed stronger anti-tumor activity than the solution, which is determined by the pharmacologic advantages of nanoassemblies: 1) extremely high drug loading (more than 80%); 2) long circulation time in the body; 3) strong ability of tumor-targeted accumulation. These factors lead to the large area of apoptosis and necrosis in tumor tissue, showing a great anti-tumor effect. In our previous study, the body weight of the nude mice after the treatment of compound **17i** at a high dose (20 mg/kg/d) was loss to some extent during the treatment (Zhang et al., 2021). In this study, H&E stained images are provided, and blood tests are conducted to demonstrate the safety of PEGylated **17i** nanoassemblies in Supplementary Figure S6, S7. This is mainly due to their biophysical targeting properties and little distribution to other tissues.

## Discussion

In the clinical setting, poorly soluble drug molecules often have low bioavailability issues and absorption problems. Thus, almost 70% of potential drugs were discarded because of their poorly soluble. As the number of poorly soluble drugs increases from discovery, developing technology to enhance their solubility as well as control their release is one of the many challenges facing the pharmaceutical industry. Reducing the size of insoluble molecules is an effective solubilization method. However, there is no simple and reliable technology to manufacture and stabilize nanoparticles in aqueous solution. Polymer carriers are used to load insoluble drug molecules, but they still face problems such as low drug loading capacity. In addition, their pharmacokinetic characteristics are closer to the properties of carrier which also affect key parameters such as distribution and drug release. Further evaluation and modification of carriers are always needed.

Carrier-free supramolecular nanoassemblies of pure compound **17i** is expected to enhance its solubility without causing problems such as low drug loading capacity as mentioned above. The drug content is greater than 80%, and the preparation process is controllable and simple. Importantly, the preparation process does not involve the use of organic solvents for dissolving hydrophobic precursors, thereby solving safety issues (such as possible toxicity) from carriers or organic solvents. On the one hand, **17i** nanoassemblies showed similar *in vitro* antitumor activity to free drugs. On the other hand, PEGylated **17i** nanoassemblies showed longer circulation times than free drugs. We speculate that its nanostructure are not substrates for some enzymes, thus the adverse metabolism of **17i** nanoassemblies caused by enzymes are reduced, which prolong the blood circulation time. In addition, we found that **17i** nanoassemblies exhibit satisfactory targeting ability, possibly due to the EPR effect. We also observed that tumors regions in C57 mice were significantly infiltrated by CD8^+^ T cells after treatment of PEGylated **17i** nanoassemblies in immunofluorescence staining and flow cytometry. Obviously, **17i** nanoassemblies elevated tumor immune response when killing tumor cells. The principle of this pure nano-drugs may open up the way to provide inspiration for maximizing the therapeutic potential of drug-like compounds.

## Conclusion

In this paper, insoluble LSD1 inhibitor, pure compound **17i**, was prepared into nanoassemblies by one-step nano-precipitation method without the addition of carrier material, and DSPE-PEG_2000_ was modified on the surface to improve stability and prolong blood circulation time. The characterization of the DLS and TEM proved that the ∼200 nm PEGylated **17i** nanoassemblies were successfully constructed. Multiple intermolecular forces in assemblies were observed using computational simulation. The PEGylated **17i** nanoassemblies had a negligible impact on the cytotoxicity of **17i** solution. As expected, the PEGylated **17i** nanoassemblies exhibited distinct advantages over **17i** solution in terms of therapeutic efficiency, anti-tumor immune response and side effects *in vivo*. This is the first time that the one-step nanoprecipitation method overcomes the problem of low water solubility of anti-tumor drug-likeness LSD1 inhibitor. Such a simple and practical nanoplatform of pure LSD1 inhibitor holds a promising application prospect for clinical cancer therapy.

## Data Availability

The original contributions presented in the study are included in the article/Supplementary Material, further inquiries can be directed to the corresponding authors.
