# Correction: Carrier-free supramolecular nanoassemblies of pure LSD1 inhibitor for effective anti-tumor therapy

**DOI:** 10.3389/fchem.2026.1816290

**Published:** 2026-05-21

**Authors:** Boao Li, Xiangyu Zhang, Jibin Li

**Affiliations:** 1 Department of Colorectal Surgery, Liaoning Cancer Hospital, Shenyang, China; 2 State Key Laboratory of Natural and Biomimetic Drugs, School of Pharmaceutical Sciences, Peking University, Beijing, China

**Keywords:** drug delivery, supramolecular nanoassemblies, therapeutic efficiency, systemic toxicity, drug-like compound

There was a mistake in Figure 7B as published. The PBS group was inadvertently duplicated from Figure 11B (Co-nanoassemblies + L group) in Sun et al. (2022). The corrected Figure 7B appears below.

**FIGURE 7:**
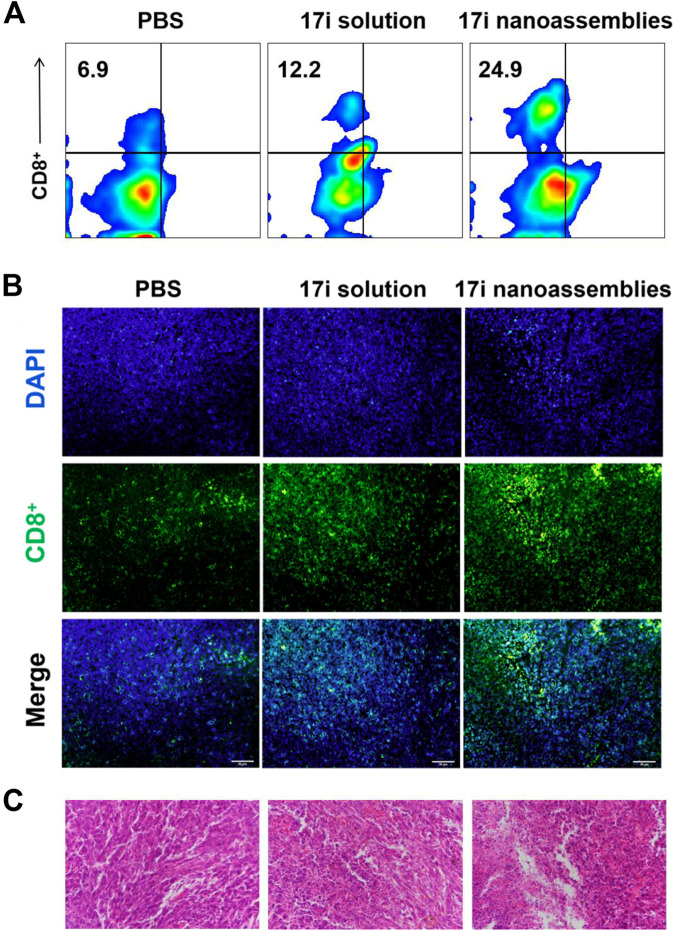
**(A)** Flow cytometry, **(B)** immunofluorescence and **(C)** H&E stained images of tumor slices. The nuclei were stained with DAPI (blue) and CD8 antibody (green). Scale bar = 50 μm.

The **Acknowledgments** statement was erroneously omitted. It reads as follows:

“The authors sincerely thank Dr. Mengchi Sun for his kind assistance with the initial immunofluorescence analysis.”

A correction has been made to the section **Materials and methods**. The subsection *Immunofluorescence experiment* was erroneously omitted. It reads as follows:

“Tumor sections were incubated in the goat serum for 1 h to reduce nonspecific background at room temperature. Next, the sections were incubated with a CD8-specific primary antibody (rabbit polyclonal antibody, 1:200, Abclonal, A0362) overnight at 4 °C. After washing, a secondary antibody (1:200, abclonal, FITC conjugation) was applied and incubated for 1 h at room temperature. The sections were then counterstained with DAPI to visualize cell nuclei. The fluorescence images were conducted using an Eclipse Ti-U inverted microscope (Nikon Corp., Tokyo, Japan).”

The original article has been updated.

