# Supplementary material for: Carrier-free supramolecular nanoassemblies of pure LSD1 inhibitor for effective anti-tumor therapy
Source: Front Chem. 2022 Sep 30;10:1012882. doi: 10.3389/fchem.2022.1012882 (PMC9561089; doi:10.3389/fchem.2022.1012882)
Supplement: Supplementary file 1 [file DataSheet1.pdf]

Supporting information

Carrier-free supramolecular nanoassemblies of pure LSD1 inhibitor for effective anti-tumor therapy

Boao Li[1], Xiangyu Zhang[2*] and Jibin Li[1*]

[1]Department of Colorectal Surgery, Liaoning Cancer Hospital, Shenyang, Liaoning, 110801, China

[2]State Key Laboratory of Natural and Biomimetic Drugs, School of Pharmaceutical Sciences, Peking University, Beijing 100191, China

***Correspondence:**

Jibin Li Ph.D

leejibin@126.com;

Xiangyu Zhang Ph.D

xiangyuzhangsyphu@163.com

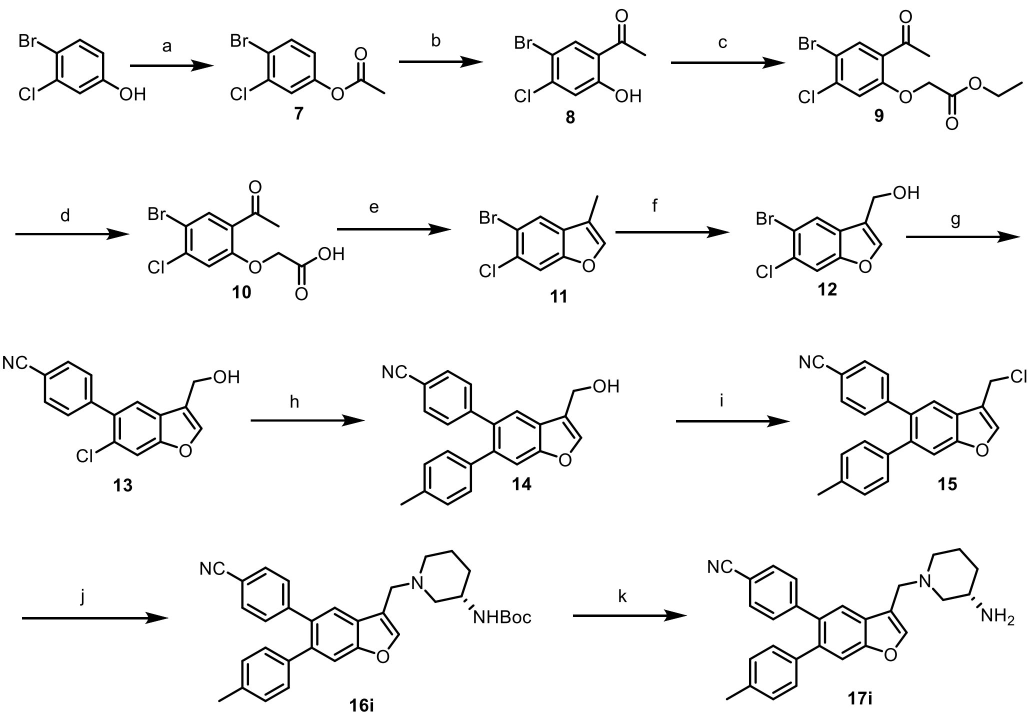

Figure S1. Synthesis of compounds **17i.** Reagents and conditions: (a): K_2CO_3, acetyl chloride, r.t. 98%; (b): $AlCl_3$, 150°C, 70%; (c): K_2CO_3, ethyl bromoacetate, 90%; (d): Na_2CO_3 aq. 100°C, 98%; (e): CH_3COOH, CH_3COONa, Acetic anhydride 120 °C, 75%; (f): SeO_2, 100°C, 85%; (g): Pd(dppf)Cl_2, 4-Cyanophenylboronic Acid, 70°C,50%; (h): Pd$(OAc)_2$, 4-Methylphenylboronic acid, 70°C, 50%; (i): $SOCl_2$, 90%; (j): tert-butyl (S)-piperidin-3-ylcarbamate, TEA, 80%; (k): HCl/EtOH, 75%.

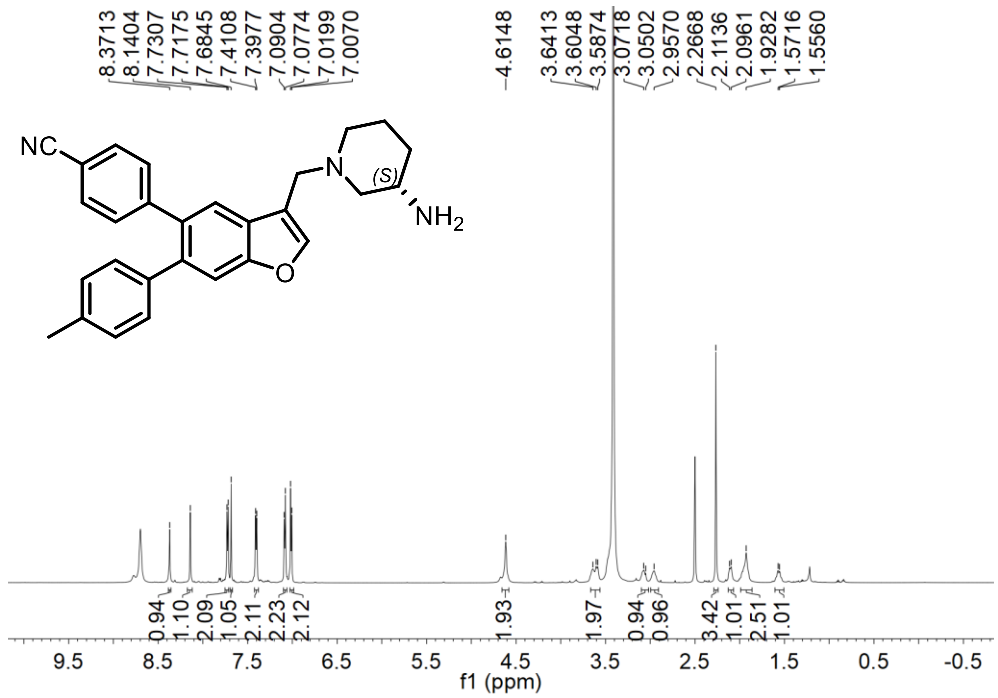

(S) - 4 - (3 - ((3-aminopiperidin-1-yl) methyl) - 6 - (p-tolyl) benzofuran-5-yl) benzonitrile (17i). Off-white solid; yield:75%; ^{1}H NMR (600 MHz, DMSO-d_6) δ 8.37 (s, 1H), 8.14 (s, 1H), 7.72 (d, J =8.0 Hz, 2H), 7.68 (s, 1H), 7.40 (d, J =7.9 Hz, 2H), 7.08 (d, J =7.8 Hz, 2H), 7.01 (d, J =7.7 Hz, 2H), 4.61 (s, 2H), 3.67 – 3.56 (m, 2H), 3.06 (d, J =13.0 Hz, 1H), 2.96 (s, 1H), 2.27 (s, 3H), 2.10 (d, J =10.5 Hz, 1H), 1.93 (s, 3H), 1.56 (d, J =9.4 Hz, 1H). ^{13}C NMR (150 MHz, DMSO-d_6) δ 155.30, 147.28, 145.27, 138.11, 137.29, 136.42, 134.08, 132.26, 131.28, 130.19, 129.19, 127.84, 122.83, 119.24, 117.82, 113.31, 109.51, 62.65, 53.61, 51.78, 48.40, 34.14, 24.25, 21.03. HRMS (EI) calcd for C28H27N3O, [M+H] $^{+}$ 422.2154, found 422.2234.

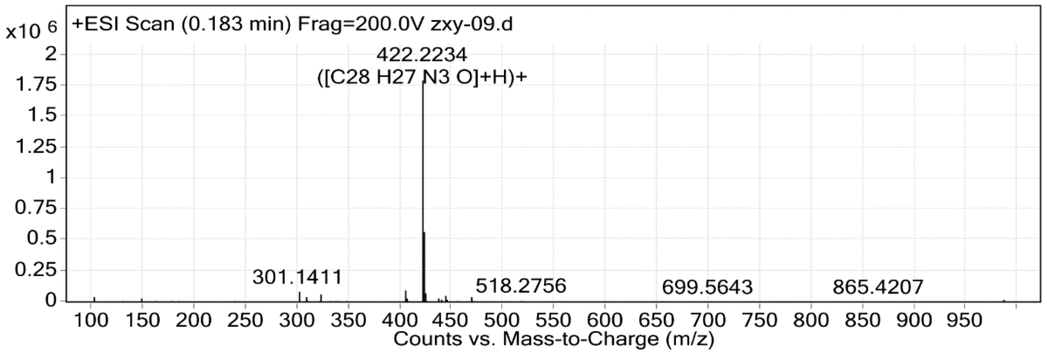

Figure S2. The ^{1}H-NMR and HR-MS spectrum of compound **17i**.

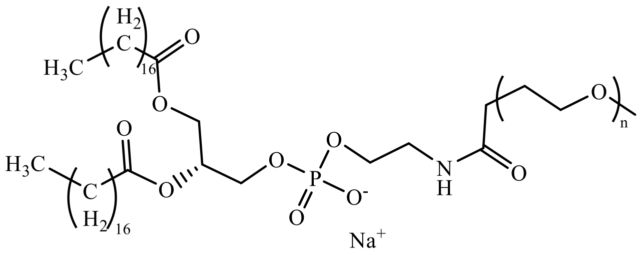

Figure S3. The chemical structure of DSPE-PEG$_{2000}$.

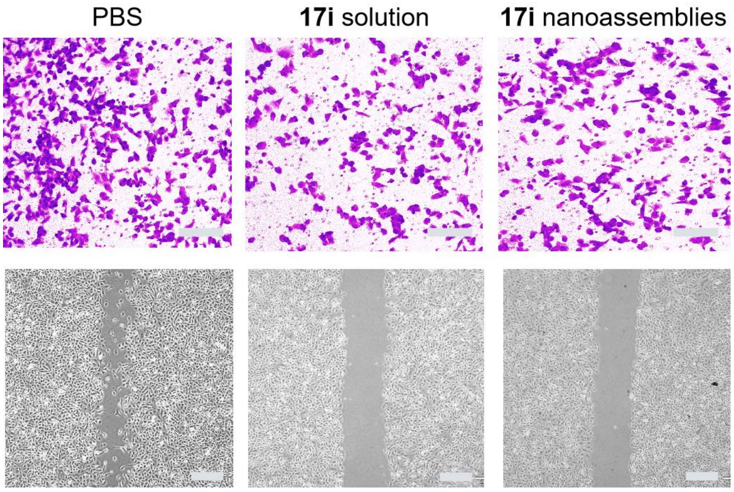

Figure S4. Typical images of up: apoptosis induction (scale bar: 50 μm) and down: wound healing assessments (scale bar: 20 μm) of PBS, **17i** solution, and **17i** nanoassemblies in CT26 cells.

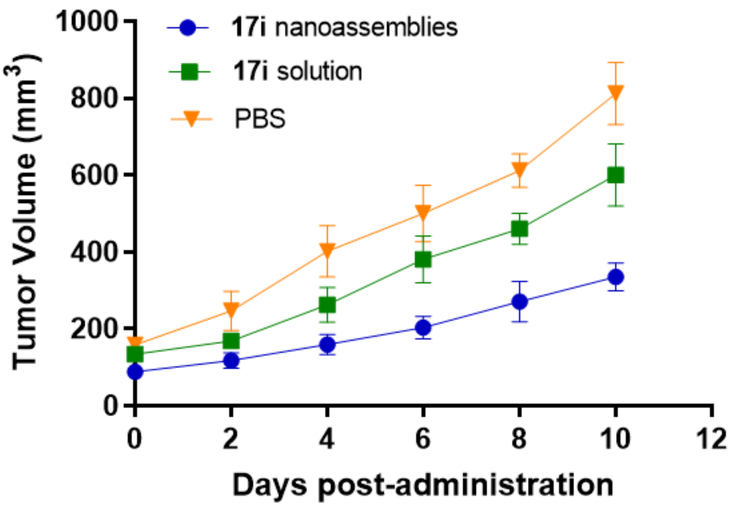

Figure S5. Therapeutic protocol on mouse CT26 subcutaneous tumor xenograft. Growth curve of the tumor volume after various treatments *via i.v*.

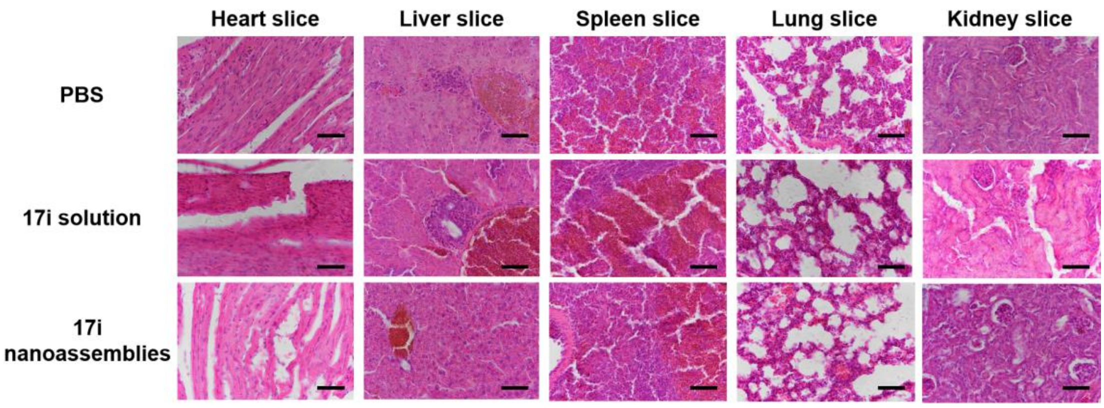

Figure S6. H&E staining images in organs after the treatment with PBS, 17i solution and 17i nanoassemblies. Scale bar=100 μm.

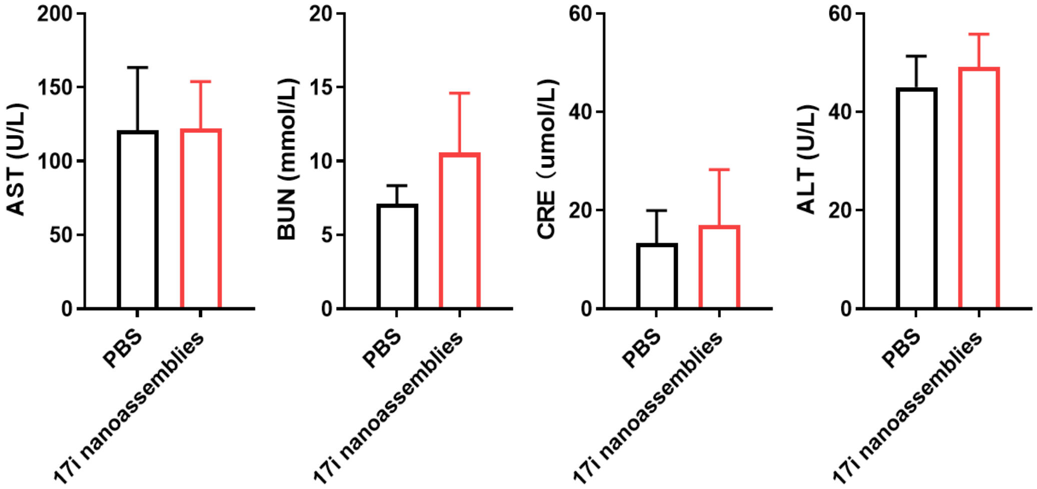

Figure S7. Quantitative analysis of blood biochemical indexes (aspartate aminotransferase (AST), blood urea nitrogen (BUN), creatinine (CRE) and alanine aminotransferase (ALT)).
